# Weight management programmes: Re‐analysis of a systematic review to identify pathways to effectiveness

**DOI:** 10.1111/hex.12667

**Published:** 2018-03-05

**Authors:** G.J. Melendez‐Torres, Katy Sutcliffe, Helen E. D. Burchett, Rebecca Rees, Michelle Richardson, James Thomas

**Affiliations:** ^1^ Division of Health Sciences Warwick Medical School University of Warwick Coventry UK; ^2^ EPPI‐Centre Social Science Research Unit UCL Institute of Education University College London London UK; ^3^ Policy Innovation Research Unit London School of Hygiene and Tropical Medicine London UK

**Keywords:** components, evidence synthesis, qualitative comparative analysis, systematic review, weight management programme

## Abstract

**Background:**

Previous systematic reviews of weight management programmes (WMPs) have not been able to account for heterogeneity of effectiveness within programmes using top‐down behavioural change taxonomies. This could be due to overlapping causal pathways to effectiveness (or lack of effectiveness) in these complex interventions. Qualitative comparative analysis (QCA) can help identify these overlapping pathways.

**Methods:**

Using trials of adult WMPs with dietary and physical activity components identified from a previous systematic review, we selected the 10 most and 10 least effective interventions by amount of weight loss at 12 months compared to minimal treatment. Using intervention components suggested by synthesis of studies of programme user views, we labelled interventions as to the presence of these components and, using qualitative comparative analysis, developed pathways of component combinations that created the conditions sufficient for interventions to be most effective and least effective.

**Results:**

Informed by the synthesis of views studies, we constructed 3 truth tables relating to quality of the user‐provider relationship; perceived high need for guidance from providers; and quality of the relationship between peers in weight management programmes. We found effective interventions were characterized by opportunities to develop supportive relationships with providers or peers, directive provider‐led goal setting and components perceived to foster self‐regulation.

**Conclusions:**

Although QCA is an inductive method, this innovative approach has enabled the identification of potentially critical aspects of WMPs, such as the nature of relationships within them, which were previously not considered to be as important as more concrete content such as dietary focus.

## 1 INTRODUCTION

In 2013, the National Institute for Health and Care Excellence (NICE) published a review on weight management programmes (WMPs).^1^ Part of this review examined how components of WMPs were associated with intervention effectiveness.^2^ However, use of external behavioural change taxonomies,^3^ or general, validated sets of recognized behavioural change techniques used across behavioural interventions, did not help to explain heterogeneity in effects in multivariate meta‐regressions. While this review was of a very high standard in conduct and reporting, the failure of an intervention taxonomy to understand effectiveness in these interventions does suggest that an alternative analytic approach could be of value in attempting to understand the “how” and “why” of intervention effectiveness. Knowing “how” and “why”—that is understanding pathways to intervention effectiveness—is important for intervention transportability and theory development, for replication and for development of adaptations to optimize interventions to the local context.

To address this gap, we aimed to understand pathways to intervention effectiveness among adult WMPs. We focused specifically on WMPs that include both diet and physical activity, as these interventions are more effective than those focusing on diet or physical activity alone,^2^ despite substantial heterogeneity. By pathways, we mean combinations of intervention features, where a combination constitutes one of possibly several ways to achieve effectiveness. Understanding pathways to high effectiveness—and to low effectiveness—can help in accounting for heterogeneity. That is to say, we aimed to understand why some interventions appeared to work better than others, or, whether specific combinations of WMP features were associated with greater intervention effectiveness. We used qualitative comparative analysis (QCA) in the service of this goal. The QCA was part of a larger project, funded by the Department of Health England, which aimed to identify the critical features of successful WMPs for adults.^4^ QCA was particularly suitable for this, as it facilitates the identification of configurations of various intervention and other contextual features that are (or are not) present when the intervention has been evaluated and found successful (or not) in obtaining a desired outcome; put otherwise, it aims to identify the necessary and sufficient conditions for achieving a desired outcome.^5^ Thus, QCA relies on a configurational understanding of causation, where a configuration of conditions can form one of multiple pathways to an outcome. As a method, QCA originated in the political science literature to develop and formalize theoretical propositions in research contexts with fewer cases than would normally be used in standard regression analysis, and where deep and comparative understandings of the included cases could guide analysis.^6^ By analogy, QCA is potentially better suited than meta‐regression and other related meta‐analytic methods for this type of analysis, as it more readily accounts for multiple “independent variables,” unlike meta‐regressions with multiple predictors, which are frequently underpowered relative to the number of regressors of interest.^5^ QCA also allows for multiple overlapping pathways to causality, and it identifies combinations of conditions as opposed to isolating the effect of one characteristic on intervention effectiveness. Thus, QCA may better represent the complex causal pathways that often characterize psychosocial interventions such as WMPs. Our QCA was informed by a synthesis of studies of user and provider views relating to WMPs.^4^ In this synthesis of views studies, which is reported in full elsewhere, we inductively analysed user views to understand important aspects of the experience of WMPs and to translate these aspects into relevant intervention features. We describe throughout this study how insights gained from this views synthesis informed each step of our analytic process.

## 2 METHODS

We examined a subset of trials evaluated in the NICE review of WMPs for overweight or obese adults^2^ that included diet, exercise and behaviour change delivered face‐to‐face compared against minimal treatment comparators. WMPs that included surgery, medication and other lifestyle changes, such as efforts at smoking cessation, were excluded. Within the 40 relevant intervention arms reported in 30 trials identified in the NICE review, we identified the ten most effective and the ten least effective interventions by examining the mean difference in weight loss between intervention and control at 12 months (from baseline) as reported for each trial. By excluding interventions shown to be moderately effective, we filtered out “noise” which might obscure differences between the most effective and least effective WMPs. Full details of trial selection are available in an Appendix S1.

In using QCA, we followed the guidance offered by Thomas, O'Mara‐Eves and Brunton (2014)^5^ in which 6 stages of analysis are outlined, covering data preparation, analysis and testing the robustness of the synthesis. As is customary, we examined pathways both to most effectiveness and to least effectiveness for each QCA model. We also focused on *sufficient* causation over *necessary* causation, as necessity is difficult and unrealistic to theorize in complex interventions. (*Necessary* causation suggests a set of conditions within which every instance of the outcome occurs, an assertion that requires strong theoretical and empirical justification, whereas *sufficient* causation suggests a set of conditions representing one of possibly several pathways to the outcome.) All analyses were undertaken using package—fuzzy—in Stata v14^7^ (StataCorp, College Station, TX).

### 2.1 Stage 1: Building the data table

We used the findings of the views synthesis^4^ to create a pre‐specified coding framework in addition to data extraction on key trial features as is standard in a systematic review (see Appendix S1, Table S1). This aimed to capture whether particular features, or in QCA terminology, “conditions,” were present or absent in the ten most and ten least effective interventions. Data were extracted by pairs of researchers who first worked independently and then compared their work to reach a consensus. After compiling data on the presence or absence of conditions for each of the interventions in a table, with the presence or absence of characteristics (or, in QCA terminology, conditions) indicated for each of the interventions, we examined the table for apparent differences between the most effective and the least effective interventions using descriptive statistics. We also reviewed the data table to check for “contradictory cases”—that is circumstances where individual conditions did not appear to discriminate clearly between most effective and least effective interventions.

### 2.2 Stage 2: Building truth tables to focus analysis on meta‐mechanisms

At this stage, the focus moved from exploring individual studies and individual conditions, as in the data table above, to exploring combinations or “configurations” of conditions and their association with either most or least effective interventions. This was done via the construction of truth tables to indicate all the possible combinations of conditions in a QCA model and how many observations in each outcome category (ie most effective or least effective interventions) correspond to that combination of conditions.

We identified a large number of possible features for inclusion in QCA models, not all of which were equally helpful in distinguishing between most effective and least effective, and some of which were of greater salience in the synthesis of views studies. To develop interpretable and meaningful analyses, we returned to our views synthesis^4^ to help in constructing more specific truth tables. The views synthesis had identified 3 important stages along the pathway to successful weight loss: attendance at WMP sessions, initial adoption of a healthier lifestyle and maintaining a healthy lifestyle. The views synthesis had also identified 3 especially salient “meta‐mechanisms,” or overarching change processes resulting from the intervention, that were perceived by study participants and authors to facilitate these stages: *social bonds* with providers or peers were perceived to motivate attendance; *accountability to others* was perceived to motivate initiation of healthy behaviours; and *experience gained through initiation of healthy behaviours* was perceived as fostering processes of self‐regulation and maintenance of healthy behaviours. As the “maintaining a healthy lifestyle” meta‐mechanism was perceived as being dependent on the earlier stages, and as both provider and peer relationships were implicated in each of the earlier stages meta‐mechanisms, 2 truth tables were explored, one with conditions anticipated to foster provider support and one with conditions anticipated to foster peer relationships.

#### 2.2.1 Provider support

The views synthesis had indicated that participants identified a need for high levels of provider support to initiate behaviour change. However, an early truth table for overall provider support that included 7 conditions was difficult to interpret and theorize in the light of the views synthesis, as no overarching interpretation emerged and results failed to explain differences between most and least effective interventions. This led us to a new line of enquiry. Through group discussion and re‐examination of the data table, we instead developed 2 separate truth tables of pathways to intervention effectiveness: one addressing provider‐user alliance (ie the degree to which the intervention enabled or intentionally fostered a relationship between provider and user) and one addressing provider directiveness (ie the degree to which interventions were characterized by provider‐led goal setting). In both of these truth tables, we additionally included conditions perceived to further support the maintenance meta‐mechanism: direct provision of exercise (perceived as facilitating experience of engaging in healthy behaviours) and graduated exit (ie intentional move from initial intensive WMP support to a more light touch approach).

#### 2.2.2 Peer relationships

Although group‐based interventions were not valued by all participants of the views synthesis, a key value of them, as identified by those who had experienced them, was that they encouraged peer relationships. The views synthesis also indicated that interventions targeted towards specific population groups enabled a “short cut” to, or increased the likelihood of, peer relationships being established in group WMPs. We further examined all these issues in 3 truth tables'. Two truth tables explored different aspects of provider support described as “alliance” and “directiveness”; one focused on peer relationships.

### 2.3 Stage 3: Checking for satisfactory spread and resolving contradictory configurations

As suggested by Thomas et al,^5^ we then examined the quality of the truth tables. We checked that there was a good spread of studies across the different configurations available within each truth table in that configurations were well represented in the interventions included in the analysis. For example, we checked that interventions were not all clustered in 4 configurations out of a possible 16 configurations of conditions.

We checked for any contradictory configurations, that is identical configurations, that are present in both most effective and least effective interventions. In relation to provider support, we found that neither of the 2 truth tables had contradictory configurations. However, in our early truth tables of peer support, we did observe contradictory configurations. This led to us pursuing several lines of enquiry. An initial truth table incorporated interventions with targeting of any kind, either population targeting or risk group targeting (ie WMPs provided to those with particular health conditions). This truth table identified several contradictory configurations as well, and as the views synthesis highlighted population group characteristics as those most likely to ensure a feeling of being with similar people, we dropped the risk group condition. We also considered the content of group work, but this did not remove contradictions. We thus took forward in our analysis the most parsimonious truth table with just 2 conditions: group work and population group targeting. This was despite contradictory configurations, which we discuss in the results.

### 2.4 Stage 4: Boolean minimization to identify the simplest expression of pathways

After checking for and resolving contradictory configurations, we used Boolean minimization to arrive at solution sets that described pathways to effectiveness. We aimed for minimized solution sets—that is the most simplified configurations—that had both complete coverage and high consistency. By “complete coverage,” we mean that when examining causal pathways to most effectiveness, we sought solutions that covered as many of the most effective studies as possible—that is that “explained” as much of the causal pathway to effectiveness as possible. By “high consistency,” we mean that we sought minimized solutions that did not also include interventions that were not most effective. By converse, when we examined causal pathways to least effectiveness, we sought minimized solutions that covered as many of the least effective studies as possible (coverage) and that did not also include most effective interventions (consistency).

### 2.5 Stage 5: Consideration of the “logical remainders” cases to understand effectiveness of hypothetical interventions

This stage of the QCA involved consideration of the hypothetical outcomes of configurations that were not present in any of the interventions. There were no logical remainders for the peer support model, but several for each of the more complex provider support models. For example, in the provider support truth table, no interventions included graduated exit alongside a lack of all of direct provisions of exercise, cultivation of provider relationships and high‐intensity provision. We consider each of these separately in the discussion.

### 2.6 Stage 6: Interpretation

In this final stage of the QCA, we interpreted the different QCA solutions in the light of the findings of the views synthesis. In an effort to ensure that the analysis accounted for our shared perspectives of the data, all conditions, configurations and models, as well as interpretations of all of these, were discussed as a group. Through this reflexive approach, we sought to justify and clearly articulate our analysis. This ensured that the final QCA models and interpretation of them were based on coherent understandings of the views synthesis.

## 3 RESULTS

We identified the 10 most effective and 10 least effective interventions collectively reported in a total of 15 trials.^8^, ^9^, ^10^, ^11^, ^12^, ^13^, ^14^, ^15^, ^16^, ^17^, ^18^, ^19^, ^20^, ^21^, ^22^ Several trials evaluated multiple interventions; 2 interventions were included from each of 3 trials^12^, ^19^, ^22^; and 3 interventions were included from a fourth.^13^ Eleven interventions involved group sessions and 16 held individual sessions; 7 included both. Ten focused on specific population or risk groups (5 focused on women^8^, ^10^, ^14^, ^19^; 2 on older adults^18^, ^21^; 2 on cardiovascular or metabolic risk^9^, ^20^; one on men only^17^) and ten did not. Eleven were high intensity,^9^, ^10^, ^11^, ^14^, ^16^, ^18^, ^19^, ^21^, ^22^ in that they had at least 48 sessions delivered at least fortnightly for at least 12 months. Interventions are described in detail in Appendix S1 (Table S2). The final conditions used in each QCA truth table and the spread of conditions across the 2 groups of interventions are displayed in Table 1. We now turn to a discussion of our findings for pathways to most effectiveness and least effectiveness demonstrated by each of the 3 QCA models. Pertinent quotes from the views synthesis are used to illustrate the underlying theory of each model.

**Table 1:** Conditions in each QCA model

Condition	Provider support models		Peer relationships model	Most effective interventions	Least effective interventions
	Provider directiveness	Provider alliance			
Direct provision of exercise	✓	✓		7	1
Provider sets energy intake	✓			10	0
Provider sets weight goals	✓			7	3
Provider sets exercise goals	✓			10	1
Provider relationships discussed		✓		10	6
Graduated exit		✓		8	2
High intensity		✓		9	2
Population group targeted			✓	8	1
Group work			✓	7	4

### 3.1 Provider‐user alliance: “You feel that somebody's batting for you”^23^

The provider‐user alliance model reflected the quality of the relationship with providers. Four conditions were included in this truth table: one reflecting the quality of the relationship (provider relationships emphasized), one reflecting opportunity for the relationship with providers to develop (high intensity) and 2 reflecting the need to move from support to self‐regulation (direct provision of exercise and graduated exit).

Each of the 4 conditions in this model discriminated between most and least effective interventions. As shown in Table 1, all ten most effective interventions emphasized provider relationships but not all least effective interventions. With regard to the other 3 conditions in this model, as shown below, their presence was more common in most effective as compared to least effective interventions. Our included interventions represented 9 of a total of 16 possible configurations, with good spread across the included interventions (see Table 2). None of the 9 configurations represented were contradictory, that is all included either most effective interventions or least effective interventions but not both.

**Table 2:** Provider directiveness truth table

Set	Most effectiveness consistency (Coverage)	Least effectiveness consistency (Coverage)	Number of cases
~Direct provision * ~provider‐set weight goals * ~provider‐set energy intake * ~provider‐set exercise goals	0.00	1.00	7
~Direct provision * provider‐set weight goals * ~provider‐set energy intake * ~provider‐set exercise goals	0.00	1.00	2
~Direct provision * provider‐set weight goals * provider‐set energy intake * provider‐set exercise goals	1.00	0.00	3
Direct provision * ~provider‐set weight goals * ~provider‐set energy intake * provider‐set exercise goals	0.00	1.00	1
Direct provision * ~provider‐set weight goals * provider‐set energy intake * provider‐set exercise goals	1.00	0.00	2
Direct provision * provider‐set weight goals * provider‐set energy intake * provider‐set exercise goals	1.00	0.00	5
~Direct provision * ~provider‐set weight goals * ~provider‐set energy intake * provider‐set exercise goals			*0*
~Direct provision * ~provider‐set weight goals * provider‐set energy intake * ~provider‐set exercise goals			*0*
~Direct provision * ~provider‐set weight goals * provider‐set energy intake * provider‐set exercise goals			*0*
~Direct provision * provider‐set weight goals * ~provider‐set energy intake * provider‐set exercise goals			*0*
~Direct provision * provider‐set weight goals * provider‐set energy intake * ~provider‐set exercise goals			*0*
Direct provision * ~provider‐set weight goals * ~provider‐set energy intake * ~provider‐set exercise goals			*0*
Direct provision * ~provider‐set weight goals * provider‐set energy intake * ~provider‐set exercise goals			*0*
Direct provision * provider‐set weight goals * ~provider‐set energy intake * ~provider‐set exercise goals			*0*
Direct provision * provider‐set weight goals * ~provider‐set energy intake * provider‐set exercise goals			*0*
Direct provision * provider‐set weight goals * provider‐set energy intake * ~provider‐set exercise goals			*0*
Provider‐set energy intake * provider‐set exercise goals * (direct provision + provider‐set weight goals)	**1.00 (1.00)**		
~Provider‐set energy intake * (~direct provision * ~provider‐set exercise goals + direct provision * ~provider‐set weight goals * provider‐set exercise goals)		**1.00 (1.00)**	

* = and, ~ = not, + = union set; italics indicate logical remainders; bold indicates reduced solutions.

**Figure 1:**
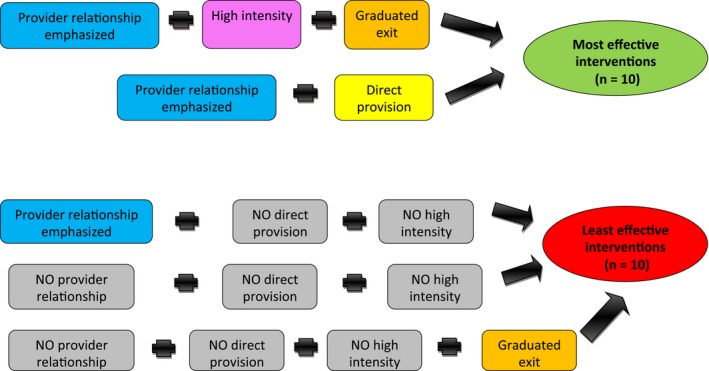
Pathways to most effectiveness and least effectiveness in the provider alliance model [Colour figure can be viewed at http://wileyonlinelibrary.com]

Our analysis revealed 2 pathways of provider support to effectiveness (see Figure 1). Both pathways were characterized by the mention of provider relationships in intervention descriptions. Additionally, most effective interventions included either direct provision of exercise or a combination of graduated exit and high intensity of provision.

Our analysis for least effectiveness was also completely consistent and covered all least effective interventions. It revealed 3 pathways to intervention least effectiveness. One pathway, which characterized 6 of the least effective interventions, revealed that interventions with provider relationships present but without both direct provisions of exercise and high intensity had reduced effectiveness. The other 2 pathways, which together accounted for the remaining 4 least effective interventions, were both characterized by a lack of emphasis on provider relationships. One pathway included a lack of direct provision of exercise and of graduated exit. The other pathway was characterized by the absence of all the other conditions in the model.

### 3.2 Provider directiveness: “I need someone to take my hand and take me over.”^24^

The provider directiveness model reflected the perceived need for a high level of guidance or direction from providers. Four conditions were included in this model: provider‐set energy intake goals, provider‐set weight goals, provider‐set exercise goals and direct provision of exercise. Each of the individual conditions included in the provider directiveness model (see Table 1) discriminated between most effective and least effective interventions. We identified interventions representing 6 of a total of 16 possible configurations in this model, with good spread across the interventions (see Table 3). None of the 6 configurations represented by the included interventions was contradictory, that is all included either most effective interventions or least effective interventions but not both.

**Table 3:** Provider‐user alliance truth table

Set	Most effectiveness consistency (Coverage)	Least effectiveness consistency (Coverage)	Number of cases
~Direct provision * ~provider relationships * ~graduated exit * ~high intensity	0.00	1.00	2
~Direct provision * ~provider relationships * ~graduated exit * high intensity	0.00	1.00	1
~Direct provision * provider relationships * ~graduated exit * ~high intensity	0.00	1.00	5
~Direct provision * provider relationships * graduated exit * ~high intensity	0.00	1.00	1
~Direct provision * provider relationships * graduated exit * high intensity	1.00	0.00	3
Direct provision * ~provider relationships * graduated exit * high intensity	0.00	1.00	1
Direct provision * provider relationships * ~graduated exit * ~high intensity	1.00	0.00	1
Direct provision * provider relationships * ~graduated exit * high intensity	1.00	0.00	1
Direct provision * provider relationships * graduated exit * high intensity	1.00	0.00	5
~Direct provision * ~provider relationships * graduated exit * ~high intensity			*0*
~Direct provision * ~provider relationships * graduated exit * high intensity			*0*
~Direct provision * provider relationships * ~graduated exit * high intensity			*0*
Direct provision * ~provider relationships * ~graduated exit * ~high intensity			*0*
Direct provision * ~provider relationships * ~graduated exit * high intensity			*0*
Direct provision * ~provider relationships * graduated exit * ~high intensity			*0*
Direct provision * provider relationships * graduated exit * ~high intensity			*0*
Provider relationships * (graduated exit * high intensity + direct provision * ~graduated exit)	**1.00 (1.00)**		
~Provider relationships * (direct provision * graduated exit * high intensity + ~direct provision * ~graduated exit) + provider relationships * ~direct provision * ~high intensity		**1.00 (1.00)**	

* = and, ~ = not, + = union set; italics indicate logical remainders; bold indicates reduced solutions.

**Figure 2:**
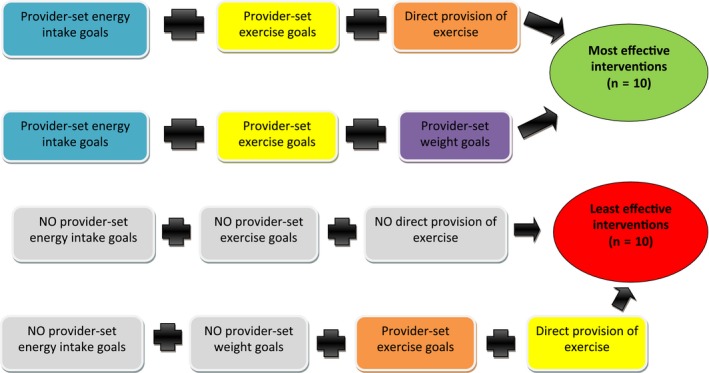
Pathways to most effectiveness and least effectiveness in the provider directiveness model [Colour figure can be viewed at http://wileyonlinelibrary.com]

Based on Table 3, we identified the simplest possible expression of configurations (see Figure 2). This identified 2 possible pathways to most effectiveness. These pathways were characterized by the presence of provider‐set energy intake goals and provider‐set exercise goals, in conjunction with the presence of either direct provision of exercise *or* provider‐set weight goals. These pathways were completely consistent (ie there were no least effective studies with these configurations) and had complete coverage (ie they represented all ten most effective interventions).

Similarly, our analysis of the provider directiveness model revealed 2 pathways to *least* effectiveness. One pathway to least effectiveness was the absence of provider‐set energy intake goals, the absence of provider‐set exercise goals and the absence of direct provision of exercise, regardless of the presence or absence of provider‐set weight goals. This pathway characterized 9 of the ten least effective interventions. An additional pathway to least effectiveness that covered the remaining least effective intervention included, alongside a lack of provider‐set energy intake goal, an absence of provider‐set weight goals even when direct provision of exercise and provider‐set exercise goals were present in the intervention.

### 3.3 Peer relationships: “You wanted to come back and hear how the guys were getting on.”^25^

The model we present for fostering of peer relationships includes 2 conditions: delivery via group sessions (a hypothesized pre‐requisite for peer relationships) and targeting a specific population group (which was perceived to enhance the likelihood of peer relationships developing in the views synthesis). We identified interventions representing all 4 possible configurations in this model, with good spread across them (see Table 4). There were no logical remainders in this truth table.

**Table 4:** Peer relationships truth table

Set	High effectiveness consistency (Coverage)	Least effectiveness consistency (Coverage)	Number of cases
~Population targeting * ~group work	0.00	1.00	5
Population targeting * ~group work	0.75	0.25	4 (3 high, 1 least)
~Population targeting * group work	0.33	0.67	6 (2 high, 4 least)
Population targeting * group work	1.00	0.00	5

* = and, ~ = not, + = union set.

Two configurations were completely consistent. All 5 interventions with population targeting and group work were most effective, and all 5 interventions without population targeting and without group work were least effective. However, both “mixed” configurations were contradictory. As described in the Methods, we pursued several lines of enquiry to resolve these contradictory configurations. However, we were unable to theorize additional reasons, and thus additional lines of enquiry, to resolve these contradictions, without departing from the data that guided these analyses.

## 4 DISCUSSION

The above analyses illustrate that fostering of supportive relationships with either providers or peers as well as provider directiveness and scaffolding for self‐regulation are potentially fundamental to the success of WMPs. While the most effective WMPs are characterized by these features, least effective WMPs are characterized by their absence. Before proceeding, we hasten to observe that each of these models must be interpreted in the light of the other; that is, the “causal recipes” for effectiveness, or ineffectiveness, from each model must be considered jointly. For example, it may not be enough for providers to be directive if they are not also developing supportive relationships with service users or providing the opportunity for group social bonds to form. Because of this, our findings provide a framework against which to understand and develop the effectiveness of WMPs. Conversely, our findings also present lessons on how *not* to implement WMPs. We hypothesize that pathways to effectiveness are characterized by a shift from extrinsic motivation to intrinsic motivation and self‐regulation. Initially, the social support they receive and a sense of accountability to providers and peers provide extrinsic motivators for service users to attend the programme and initiate healthy behaviours. With this support, service users are able to experience their ability to participate in exercise or eat healthily, and over time these experiences enable them to realize that they are capable of behaviour change and as they experience its benefits, participants develop intrinsic motivation. This intrinsic motivation encourages self‐regulation and maintenance of behaviour change. Without initial intensive support in the pathway, through provider support and directiveness, or peer relationships, WMPs are unlikely to unlock the intrinsic motivation necessary for successful self‐regulated weight management.

QCA models are an example of “abductive” reasoning oriented towards building theory. That is, they blend inductive reasoning of the sort that characterizes qualitative research, especially forensic knowledge of included cases, and deductive reasoning, or hypothesis testing, of the sort that characterizes statistical inference and hypothesis testing.^26^ QCA is shaped by an understanding of relevant theory, which in this case was supplied by the views synthesis. Because of this, our findings should not be viewed as conclusive but rather as developing a middle‐range theory, or “inference to the best explanation,” of how WMPs can achieve weight loss outcomes. Weiss (1997) outlines 2 components to an intervention's theory of change: an “implementation theory,” or a statement about what needs to be undertaken in implementing a programme and a “programmatic theory,” which intends to describe *how* interventions work.^27^ Because we focused here on *what* was being done in interventions as well as *how*, our findings blend both types of theory. We will now summarize each of our models and attend to logical remainders (ie configurations of conditions that were not actually present in included interventions) that arose in the analysis.

### 4.1 Provider‐user alliance

Provider relationships appear to be a necessary condition for most effectiveness (ie the most effective interventions were a subset of interventions describing provider relationships), but on their own provider relationships are not sufficient for most effectiveness. Interventions also need to include a mechanism for encouraging self‐regulation, either via direct provision of exercise or via an intentionally graduated reduction in support after an initial more intensive period. Absence of either provider relationships or conditions that foster self‐regulation will result in reduced effectiveness.

We identified 7 possible configurations in the provider alliance model that were not present in any of the included interventions, of which we judged that 6 would lead to least effectiveness, either because they lacked provider relationships or because they did not foster self‐regulation via direct exercise provision. Throughout our views synthesis, and later in our QCA models, a theme that emerged was the centrality of relationship with the intervention providers as critical to successful weight management. However, one logical remainder, including direct provision of exercise, provider relationships and graduated exit, is likely to be effective because the combination of provider relationships and direct provision of exercise would, based on our views synthesis, creates the conditions for continued exercise and weight management.

### 4.2 Provider directiveness

The most effective interventions involved a high level of direction from providers, including provider‐set behavioural directives addressing both energy intake and energy expenditure. Absence of either a provider‐set energy intake goal or a provider‐set energy expenditure was part of the causal recipe for least effectiveness.

Based on our analysis, we believe that most of the logical remainders in our provider directiveness model are more likely to lead to least effective interventions than most effective interventions. As discussed earlier, the views synthesis revealed that interventions should initially provide a high level of support and directiveness but also incorporate some conditions which foster self‐regulation to move from external motivation to internal. The only condition in this model perceived as fostering self‐regulation is direct provision of exercise; thus, we believe that all 5 remaining configurations without this condition would likely be least effective. Moreover, as our analyses found that directive energy intake goals were present in both pathways to effectiveness and absent in both pathways to least effectiveness, we conclude that remaining configurations without this condition would also be a recipe for least effectiveness. We did not believe that provider‐set weight goals would be sufficient as either an intake or expenditure requirement, and indeed participants in some of the studies analysed in our views synthesis described “gaming” weight loss goals by, for example, using a sauna before weigh‐ins. However, it is possible that the remaining 2 logical remainders, combining direct provision of exercise with provider‐set energy intake goals, could be effective.

### 4.3 Peer relationships

The combination of group delivery, which provided an opportunity for developing peer relationships, and population targeting was a recipe for most effectiveness, whereas interventions lacking both of these conditions were least effective. Our views synthesis reflected the importance of group identification and social process as fostering commitment to the WMP.

As noted above, we were unable to theorize the contradictory “intermediate” configurations based on the views synthesis. However, drawing on the views synthesis, we concluded that the presence of both conditions ensures that the full value of each is “unlocked” as follows. The presence of group work *alone* may encourage peer relationships, which was perceived to increase likelihood of WMP attendance (through social bonds) and initiation of healthy behaviours (through peer accountability). Moreover, while the presence of population targeting *alone* would help to ensure the delivery of appropriate services meeting user needs, evidence from the views synthesis indicated that when population targeting is present *in conjunction* with delivery to groups, it created a short cut to beneficial peer relationships because of the presence of similar others in the group. Thus, if population targeting is present on its own, only one of its 2 beneficial mechanisms are unlocked, and if group work is present on its own, the likelihood of peer relationships forming is diminished.

### 4.4 In relationship to prior literature

Our QCA models extend what is by now a classic theoretical model whereby goal setting is key to the development of self‐regulation. Specifically, as regards to weight management, our QCA models suggest that provider‐led goal setting is essential to the development of this self‐regulation. Moreover, Burke, Wang and Sevick's systematic review^28^ have documented the importance of self‐monitoring to promoting weight loss in WMPs, as did Michie and colleagues in physical activity and healthy eating interventions.^29^ Michie and colleagues also found that combining self‐monitoring with additional techniques was associated with even stronger effects. Although we did not have the data to explore self‐monitoring specifically in our QCA, it is possible that provider‐led goal setting worked in part as a means of having something to self‐monitor. Our analysis nuances these previous reviews' findings by suggesting that self‐monitoring may be usefully employed in the context of provider‐led goal setting and that combining self‐monitoring with this provider‐led goal setting could be an important “tipping point” to most effective interventions. In addition to confirming the relevance of self‐monitoring, Dombrowski and colleagues^30^ found that number of components was not associated with effectiveness. Our analysis complements the authors' conclusion by noting that the combination of components may be more important than their quantity.

What is interesting, however, is that our findings contradict the theoretical proposition, originating from self‐determination theory, that intrinsic motivation via self‐authored goals is key to the success of behaviour change.^31^, ^32^ Instead, our findings suggest that carefully structured extrinsic motivation, via provider‐led goals, is key to effectiveness. Another perspective on self‐determination theory is that extrinsic, but “active,” motivation can be of utility in achieving goals.^32^ Because our findings suggested the importance of both accountability to others via group social bonds and provider relationships, it is possible that WMPs following the pathways to effectiveness developed here generate the type of extrinsic motivation necessary to “jumpstart” the development of intrinsic motivation. Although we were unable to test temporality in this analysis, findings from the views synthesis suggested that the creation of this “active” extrinsic motivation led to self‐regulation later on. For example, as 1 participant noted about the WMP, supervised exercise created an “appetite” for more exercise later on: *“It's got me going back to the gym and stuff like that, on top of the walking”*.^33^

### 4.5 In relation to the previous review

Our analysis tested some of the same conditions as the original NICE review.^2^ In the original analysis presented to NICE, neither direct provision of exercise (what they label “supervised exercise”) nor graduated exit were useful in explaining heterogeneity in effects to statistical significance, although provider‐set energy intake goals (labelled in their review as “set energy prescription”) were associated with increased weight loss. Our findings are not strictly comparable, in that we used Boolean logic to theorize causal combinations rather than the “net effects” approach that multivariate meta‐regression would suggest. However, our QCA models were able to describe the role of direct provision of exercise and graduated exit in pathways to intervention effectiveness; additionally, we were able to describe how provider‐set energy intake goals were important for effectiveness in combination with other conditions. We were able to replace an etic (ie inductively and internally derived) intervention component taxonomy with an emic one that was driven by the views of service users and providers. While broadly generalizable taxonomies are important for making sense of heterogeneous interventions, we believe our approach has the capability to look from “within” interventions at the differences users and providers would find salient.

### 4.6 Strengths and limitations

Conditions were coded independently and in duplicate (by KS, HEDB and MR), as is consistent with rigorous systematic review methods. We also began by grounding our analysis closely in the views synthesis and kept an auditable record of analytic decisions along the way. This is a strength for 2 reasons. First, it lent structure to what is in part an inductive method of analysis and thus avoided the problem of data fishing. Second, it meant that the voices and perspectives of service users were “carried through” the analysis. Even where our analysis was guided by our reading of the trials—for example, our decision to split provider support into alliance and directiveness—we were able to relate and interpret our decision to consider directiveness separately from alliance through the findings included in our views synthesis.

However, this analysis has several limitations as well. Incorporation of study quality assessments in QCA is not as yet well understood, and thus, we were unable to consider study quality in our models. But, perhaps counterintuitively, trials supplying “most effective” interventions were of higher quality than trials supplying “least effective” interventions as judged by the original review group.^1^ Poor reporting of intervention content means that deeper engagement with intervention conditions was difficult. For example, poor intervention reporting on provider support meant that we needed to consider a variety of “cues” in intervention descriptions to understand whether provider support was present. Access to original intervention protocols or study manuals could have facilitated coding of interventions, as would have clearer descriptions of interventions in included trials. Although our decision to exclude trials of “middling” effectiveness meant that we were able to theorize and focus directly on the most potent comparisons, we may have missed important information by setting those trials aside. Additionally, we were unable to explore potentially interesting conditions in our analysis because of a lack of studies including them. Several conditions, such as programme flexibility, diet‐related components and delivery format, were present in few interventions across both most effective and least effective ones (n ≤ 5/20), and their relevance for weight management is difficult to establish. Finally, while interventions spread well over configurations of causal conditions for which we had cases, we did have several logical remainders in 2 of our models.

## 5 CONCLUSIONS

In our analysis, pathways to intervention effectiveness were characterized by the presence of opportunities to develop supportive relationships with providers or peers, clear direction from providers together with components that foster the development of self‐regulation. We hypothesize that supportive relationships garnered extrinsic motivation, which encouraged attendance and initiation of behaviour change, which in turn helped to develop intrinsic motivation, which encouraged self‐regulation and maintenance of behaviour change. QCA analyses are by their nature inductive and tentative, and other pathways are possible as well, or may become apparent as more research accumulates. However, this innovative approach has suggested the importance of potentially critical aspects of WMPs, such as the nature of relationships within them, which were previously not considered to be as important as more concrete content such as dietary focus. By setting out the mechanisms through which effectiveness could be achieved, as well as the conditions such mechanisms require, these findings should prove useful for interventionists, commissioners and others considering implementing a WMP.

## COMPETING INTERESTS

Authors have no competing interests to declare.
