# Supplementary material for: Weight management programmes: Re‐analysis of a systematic review to identify pathways to effectiveness
Source: Health Expect. 2018 Mar 5;21(3):574–84. doi: 10.1111/hex.12667 (PMC5980502; doi:10.1111/hex.12667)
Supplement: Supplementary file 1 [file HEX-21-574-s001.docx]

**Online table 1.** Coding framework as applied to included interventions.

| **Domain** | **Intervention features** | **Highly effective interventions** | | | | | | | | | | **Less effective interventions** | | | | | | | | | | **Total highly effective (n=10)** | **Total less effective (n=10)** |
| --- | --- | --- | --- | --- | --- | --- | --- | --- | --- | --- | --- | --- | --- | --- | --- | --- | --- | --- | --- | --- | --- | --- | --- |
|  |  | **Bertz 2012** | **DPP 2002** | **Foster-Schubert 2012** | **Kuller 2012** | **Rejeski 2011** | **Rock 2010 (CB)** | **Rock 2010 (TB)** | **Villareal 2011** | **Vissers 2010 (fitness)** | **Vissers 2010 (vibration)** | **Eriksson 2009** | **Hersey 2012 (2)** | **Hersey 2012 (3)** | **Jolly 2011 (GP)** | **Jolly 2011 (pharmacist)** | **Jolly 2011 (SW)** | **Munsch 2003 (clinic)** | **Nanchahal 2011** | **Patrick 2011** | **Vermunt 2011** |  |  |
| **1. Provider support** | Provider-user relationship emphasised | 1 | 1 | 1 | 1 | 1 | 1 | 1 | 1 | 1 | 1 | 0 | 0 | 1 | 1 | 1 | 1 | 0 | 1 | 0 | 1 | **10** | **6** |
|  | Provider role: interaction for individualised support | 1 | 1 | 1 | 1 | 1 | 1 | 1 | 1 | 1 | 1 | 0 | 0 | 1 | 1 | 1 | 1 | 0 | 0 | 0 | 1 | **10** | **5** |
|  | Provider manner: encouraging/supportive | 1 | 1 | 0 | 1 | 1 | 0 | 0 | 0 | 0 | 0 | 0 | 0 | 1 | 0 | 0 | 1 | 0 | 1 | 0 | 0 | **4** | **3** |
|  | Dietician | 1 | 1 | 1 | 1 | 0 | 0 | 0 | 1 | 1 | 1 | 1 | 0 | 0 | 0 | 0 | 0 | 0 | 0 | 1 | 1 | **7** | **3** |
| **2. Addressing user needs and preferences** | Risk group targeting | 0 | 0 | 0 | 0 | 0 | 0 | 0 | 0 | 0 | 0 | 1 | 0 | 0 | 0 | 0 | 0 | 0 | 0 | 0 | 1 | **0** | **2** |
|  | Population group targeting | 1 | 1 | 1 | 1 | 1 | 1 | 1 | 1 | 0 | 0 | 0 | 0 | 0 | 0 | 0 | 0 | 0 | 0 | 1 | 0 | **8** | **1** |
|  | Flexible programme | 0 | 1 | 0 | 0 | 0 | 0 | 0 | 0 | 0 | 0 | 0 | 0 | 0 | 0 | 0 | 0 | 0 | 0 | 0 | 0 | **1** | **0** |
| **3. Monitoring** | Weight monitoring | 1 | 1 | 1 | 0 | 1 | 0 | 0 | 1 | 0 | 0 | 0 | 1 | 1 | 1 | 1 | 1 | 0 | 0 | 0 | 0 | **5** | **5** |
|  | Private weight monitoring | 1 | 1 | 0 | 0 | 1 | 0 | 0 | 0 | 0 | 0 | 0 | 1 | 1 | 1 | 1 | 1 | 0 | 0 | 0 | 0 | **3** | **5** |
|  | Diet monitoring | 1 | 0 | 1 | 1 | 1 | 0 | 0 | 1 | 0 | 0 | 0 | 1 | 1 | 1 | 1 | 1 | 1 | 1 | 0 | 1 | **5** | **8** |
|  | Diet monitoring made 'easy' | 0 | 1 | 0 | 0 | 0 | 0 | 0 | 0 | 0 | 0 | 0 | 0 | 0 | 0 | 0 | 0 | 0 | 0 | 1 | 0 | **1** | **1** |
|  | Exercise monitoring | 1 | 1 | 1 | 1 | 1 | 0 | 0 | 0 | 1 | 0 | 1 | 1 | 1 | 0 | 0 | 0 | 1 | 1 | 1 | 0 | **6** | **6** |
| **4. Delivery** | Face-to-face | 1 | 1 | 1 | 1 | 1 | 1 | 0 | 1 | 1 | 1 | 1 | 0 | 0 | 1 | 1 | 1 | 1 | 1 | 0 | 1 | **9** | **7** |
|  | Remote | 1 | 0 | 1 | 0 | 0 | 1 | 1 | 0 | 0 | 0 | 0 | 1 | 1 | 0 | 0 | 1 | 0 | 0 | 1 | 0 | **4** | **4** |
|  | Group sessions | 0 | 1 | 1 | 1 | 1 | 0 | 0 | 1 | 1 | 1 | 1 | 0 | 0 | 0 | 0 | 1 | 1 | 0 | 0 | 1 | **7** | **4** |
|  | Individual sessions | 1 | 1 | 1 | 0 | 1 | 1 | 1 | 0 | 1 | 1 | 0 | 1 | 1 | 1 | 1 | 1 | 0 | 1 | 1 | 1 | **8** | **8** |
|  | High intensity | 0 | 1 | 1 | 1 | 1 | 1 | 1 | 1 | 1 | 1 | 1 | 0 | 0 | 0 | 0 | 0 | 0 | 1 | 0 | 0 | **9** | **2** |
| **5. Diet** | Practical diet information | 1 | 1 | 0 | 1 | 1 | 1 | 1 | 0 | 0 | 0 | 1 | 0 | 0 | 1 | 1 | 1 | 0 | 1 | 1 | 1 | **6** | **7** |
|  | Visual demonstrations | 1 | 0 | 0 | 0 | 1 | 0 | 0 | 0 | 0 | 0 | 0 | 0 | 0 | 1 | 1 | 1 | 0 | 0 | 0 | 0 | **2** | **3** |
|  | De-emphasise diet | 0 | 0 | 0 | 0 | 0 | 0 | 0 | 0 | 0 | 0 | 0 | 0 | 0 | 0 | 0 | 0 | 0 | 0 | 0 | 0 | **0** | **0** |
| **6. Exercise** | Supervised exercise provision | 1 | 1 | 1 | 0 | 1 | 0 | 0 | 1 | 1 | 1 | 1 | 0 | 0 | 0 | 0 | 0 | 0 | 0 | 0 | 0 | **7** | **1** |
|  | Focus on fitness gains | 1 | 1 | 1 | 0 | 0 | 0 | 0 | 1 | 0 | 0 | 1 | 0 | 0 | 0 | 0 | 0 | 0 | 1 | 1 | 0 | **4** | **2** |
|  | Tailored to fitness levels | 1 | 0 | 1 | 1 | 0 | 1 | 1 | 0 | 1 | 1 | 0 | 1 | 1 | 0 | 1 | 1 | 0 | 1 | 1 | 0 | **7** | **6** |
|  | Graduated intensity | 1 | 0 | 1 | 1 | 1 | 0 | 0 | 1 | 1 | 1 | 1 | 0 | 0 | 1 | 1 | 1 | 1 | 0 | 1 | 0 | **7** | **6** |
|  | Highlight services available | 0 | 0 | 0 | 0 | 0 | 0 | 0 | 0 | 0 | 0 | 1 | 0 | 0 | 0 | 0 | 1 | 0 | 1 | 0 | 0 | **0** | **3** |
| **7. Goals** | Provider-set energy-intake goal | 1 | 1 | 1 | 1 | 1 | 1 | 1 | 1 | 1 | 1 | 0 | 0 | 0 | 0 | 0 | 0 | 0 | 0 | 0 | 0 | **10** | **0** |
|  | User identified diet goal | 0 | 0 | 0 | 0 | 0 | 0 | 0 | 0 | 0 | 0 | 0 | 0 | 0 | 1 | 1 | 1 | 1 | 0 | 1 | 0 | **0** | **5** |
|  | Provider-set weight goal | 1 | 1 | 1 | 1 | 1 | 1 | 1 | 1 | 0 | 0 | 0 | 0 | 0 | 1 | 1 | 0 | 0 | 0 | 0 | 0 | **8** | **2** |
|  | User identified weight goal | 0 | 0 | 0 | 0 | 0 | 0 | 0 | 0 | 0 | 0 | 1 | 0 | 0 | 0 | 0 | 1 | 1 | 0 | 1 | 0 | **0** | **4** |
|  | Provider-set exercise goal | 1 | 1 | 1 | 1 | 1 | 1 | 1 | 1 | 1 | 1 | 1 | 0 | 0 | 0 | 0 | 0 | 0 | 0 | 0 | 0 | **10** | **1** |
|  | User identified exercise goal | 0 | 0 | 0 | 0 | 0 | 0 | 0 | 0 | 0 | 0 | 1 | 0 | 0 | 1 | 1 | 1 | 1 | 0 | 1 | 0 | **0** | **6** |
| **8. External moderators** | Problem solving, relapse prevention | 1 | 1 | 1 | 1 | 1 | 1 | 1 | 0 | 0 | 0 | 1 | 1 | 1 | 1 | 1 | 1 | 1 | 1 | 0 | 1 | **7** | **9** |
|  | Individualised problem solving, relapse prevention | 1 | 1 | 1 | 0 | 1 | 0 | 0 | 0 | 0 | 0 | 1 | 0 | 1 | 1 | 1 | 0 | 0 | 1 | 0 | 1 | **4** | **6** |
| **9. Follow-on** | Graduated exit | 0 | 1 | 1 | 1 | 1 | 1 | 1 | 0 | 1 | 1 | 1 | 0 | 0 | 0 | 0 | 0 | 0 | 1 | 0 | 0 | **8** | **2** |
| **10. Other** | Competition emphasised | 0 | 1 | 0 | 0 | 0 | 0 | 0 | 0 | 0 | 0 | 0 | 0 | 0 | 0 | 0 | 0 | 0 | 0 | 0 | 0 | **1** | **0** |
|  | Social benefits emphasised | 0 | 0 | 1 | 1 | 1 | 0 | 0 | 0 | 0 | 0 | 1 | 1 | 1 | 0 | 0 | 1 | 0 | 0 | 1 | 1 | **3** | **6** |
|  | Mental-wellbeing emphasised | 0 | 0 | 0 | 0 | 1 | 0 | 0 | 0 | 0 | 0 | 0 | 0 | 0 | 0 | 0 | 0 | 0 | 0 | 0 | 0 | **1** | **0** |
|  | Fun emphasised | 0 | 0 | 0 | 0 | 0 | 0 | 0 | 0 | 0 | 0 | 0 | 0 | 0 | 0 | 0 | 0 | 0 | 1 | 0 | 0 | **0** | **1** |

**Online table 2.** Included interventions.

| **Study ID (arm)** | **Study aim** | **Country** | **n (focal intervention and control)** | **Study recruitment** | **Comparator** | **Study population** | **Mean age of participants** | **% female** | **% ethnic minority** | **% Some college education** | **Difference (kg) at 12 mos [95% CI]** |
| --- | --- | --- | --- | --- | --- | --- | --- | --- | --- | --- | --- |
| **Most effective interventions** | | | | | | | | | | | |
| **Bertz 2012** | Weight loss | Sweden | 33* | PC | NI | Breastfeeding women | 34 | 100% | 3 | 69% | -6.00 [-8.05, -3.95] |
| **DPP 2002** | Diabetes prevention | USA | 2161 | MR | SSMO | US ethnic minority populations | 51 | 68% | 54 | 74% | -6.10 [-6.65, -5.55] |
| **Foster-Schubert 2012** | Weight loss | USA | 204* | MR | NI | Post-menopausal women | 58 | 100% | 15 | 70% | -8.20 [-9.59, -6.81] |
| **Kuller 2012** | Slow subclinical atherosclerosis in women on HRT | USA | 508 | MR | SSOther | Post-menopausal women | 57 | 100% | 12 | 80% | -5.10 [-6.18, -4.02] |
| **Rejeski 2011** | Increased mobility | USA | 191* | MR | SSOther | Older adults with poor cardiovascular health | 67 | 67% | 15 | 47% | -5.50 [-7.61, -3.39] |
| **Rock 2010 (centre-based)** | Weight loss | USA | 278* | MR | SSMO | Women only | 44 | 100% | 32 | 74 | -7.60 [-9.57, -5.63] |
| **Rock 2010 (telephone-based)** | Weight loss | USA | 275* | MR | SSMO | Women only | 44 | 100% | 21 | 73 | -6.00 [-8.05, -3.95] |
| **Villareal 2011** | Weight loss and improved physical function | USA | 55* | MR | SSMO | Aged 65 years or older | 70 | 63% | NR | 68% | -7.80 [-9.84, -5.76] |
| **Vissers 2010 (fitness)** | Weight loss | Belgium | 41* | PC | NI | Non-targeted | 45 | 74.7 | NR | NR | -7.40 [-10.85, -3.95] |
| **Vissers 2010 (vibration)** | Weight loss | Belgium | 36* | PC | NI | Non-targeted | 43 | 74.7 | NR | NR | -8.30 [-11.99, -4.61] |
| **Least effective interventions** | | | | | | | | | | | |
| **Eriksson 2009** | CVD prevention | Sweden | 151 | PC | DAC | Cardiovascular risk | 54 | 57% | NR | NR | -0.60 [-1.45, 0.25] |
| **Hersey 2012 (2)** | Weight loss | USA | 1177* | MR | DAC | Non-targeted | 47 | 71.8 | 5.5 | NR | -0.70 [-1.37, -0.03] |
| **Hersey 2012 (3)** | Weight loss | USA | 1176* | MR | DAC | Non-targeted | 47 | 77 | 5.9 | NR | -0.60 [-1.28, 0.08] |
| **Jolly 2011 (GP)** | Weight loss | UK | 170* | PC | NI | Non-targeted | 50 | 67% | 10 | NR | 0.30 [-2.47, 3.07] |
| **Jolly 2011 (pharmacist)** | Weight loss | UK | 170* | PC | NI | Non-targeted | 49 | 73% | 13 | NR | 0.40 [-2.31, 3.11] |
| **Jolly 2011 (SW)** | Weight loss | UK | 200* | PC | NI | Non-targeted | 49 | 65% | 12 | NR | -0.80 [-3.42, 1.82] |
| **Munsch 2003 (clinic)** | Weight loss | Switzerland | 69* | PC | SSMO | Non-targeted | 46 | 75% | NR | NR | -0.70 [-3.35, 1.95] |
| **Nanchahal 2011** | Weight loss | UK | 381 | PC | NI | Non-targeted | 49 | 72% | 29 | NR | -0.30 [-1.18, 0.58] |
| **Patrick 2011** | Weight loss | USA | 441 | MR | NI | Men only | 44 | 0% | 29 | 69 | -0.70 [-1.96, 0.56] |
| **Vermunt 2011** | Diabetes prevention | Netherlands | 764* | PC | DAC | Risk of developing type 2 diabetes | 58 | 60% | NR | NR | -0.20 [-0.82, 0.42] |

* Study reports additional trial arms that were not included in the analysis

MR = Mass recruitment (direct mailing/advertisement); PC = Personalised contact via clinic/GP

NI = No intervention or leaflet(s) only; DAC = Discussion/advice/counselling in one-off session +/- leaflet; SSMO = Seeing someone more than once for weight management, provider untrained +/- leaflets; SSOther = Seeing someone more than once for discussion other than weight loss
