# Supplementary material for: Weight management programmes: Re‐analysis of a systematic review to identify pathways to effectiveness
Source: Health Expect. 2018 Mar 5;21(3):574–84. doi: 10.1111/hex.12667 (PMC5980502; doi:10.1111/hex.12667)
Supplement: Supplementary file 2 [file HEX-21-574-s002.docx]

**Study identification methods**

Because the weight management and obesity literatures have been extensively reviewed, we aimed to focus resource on analysis rather than study identification.

**Identification of trials**

We identified trials of WMPs shown to be highly effective for achieving weight loss among people with obesity and WMPs shown to have lower effects in the NICE (2013) review^1^. This approach, similar to maximum variation sampling typically employed in qualitative research, and MSDO/MDSO (most similar, different outcome/most different, similar outcome) designs ^2^ was used to enhance our ability to detect the critical features of successful WMPs. By excluding interventions shown to be moderately effective, we filtered out ‘noise’ which might obscure differences between the highly effective and less effective WMPs. We identified the ten most effective and the ten least effective interventions evaluated in the NICE review, in terms of the mean difference in weight loss between intervention and control at 12 months (from baseline). In one of the least effective interventions ^3^ the control group unexpectedly lost a lot of weight, which called into question the reliability of the study findings. We therefore excluded this study and selected the next least effective to ensure that we had ten in each group. Because study quality is not well understood in the context of QCA, we did not otherwise specifically select trials on the basis of study quality.

**Inclusion criteria**

Trials evaluating the selected interventions all met the inclusion criteria set out in the NICE (2013) review ^4^. Studies were all RCTs of WMPs for adults (≥ 18 years) classified as overweight or obese, i.e. people with a BMI of ≥ 25 kg/m2 and ≥ 30 kg/m2, respectively, or a BMI of ≥ 23 kg/m2 in Asian populations. The intervention had to contain a combination of diet and exercise with a behaviour change strategy to influence lifestyles and be delivered in the health sector, in the community or commercially. Included WMPs assessed weight loss at follow-up of 12 months or more. Included comparators were no intervention at all or leaflet/s only; discussion/advice/counselling in one-off session +/-leaflet; seeing someone more than once for discussion of something other than weight loss and seeing someone more than once for weight management, person untrained +/- leaflets. WMPs that included surgery, medication and other lifestyle changes such as efforts at smoking cessation were excluded. For full details please refer to the NICE (2013) review ^4^.

**Quality assessment**

We used the study quality scores as apprised in the NICE review ^4^ which was based on the York CRD approach^5^ as described in the CPHE Methods Manual, but did not evaluate on the basis of blinding. Overall scores of internal and external validity were generated and graded as ++ (most of checklist criteria were fulfilled and conclusions were judged very unlikely to alter), + (some criteria were fulfilled and conclusions were unlikely to alter) or – (few or no criteria were fulfilled and conclusions were likely or very likely to alter) for each study. Internal validity was based on assessment of the randomisation and allocation procedures, evidence of selective reporting and attrition. External validity was based on how representative the study sample is of the general population and how applicable the findings are to implementation in the UK.

**Data extraction**

To extract information about the features of the selected WMP interventions we developed a coding framework based on the findings of the views synthesis. Data were extracted by two researchers who first worked independently and then compared their work to reach a consensus.

We developed the coding framework with the intention of reflecting the key features and domains of WMPs as identified in the views synthesis. As such the framework, presented in Supplementary File 2 Online Table 1, reflects each of the seven programme domains identified, as key as well as reflecting views about external moderators and programme follow-on.

Capturing information about intervention characteristics was not always straight-forward; often there was little detail. For example, provider support, arguably the most significant intervention feature according to users and providers in the views syntheses, was rarely described in any detail, thus we inferred a level of provider relationship, for example, that interventions involving counselling would incorporate some level of provider-user relationships. Despite these challenges, we applied the coding framework to each of the interventions, capturing evidence for each of the characteristics and assigning interventions to the relevant ‘conditions’.

We also utilised additional data including the methods of recruitment used and variables for other intervention characteristics reported in the NICE (2013) review ^4^ which are summarised in the table below. Changes to some of the codes that NICE applied were modified where extraction errors were identified.

**Methodological information extracted from the NICE (2013) review**

| **Program feature** | **Information included** |
| --- | --- |
| Bibliographic & study details | Study aim  Country  Sample size  Study recruitment method |
|  | Control group |
| Participant characteristics | Population group targeted  Mean age (years)  Percentage of female participants  % ethnic minority  % some college education |
| Effective intervention components found by NICE | Dietician  Energy intake prescription (set energy prescription) |
| Weight outcomes | Outcomes at 12, 18, and 36 months |
| Risk of bias | Assessed using the York CRD approach^5^ |

1. Hartmann-Boyce J, Johns D, Aveyard P, et al. *How components of behavioural weight management programmes affect weight change: Review 1b.* London: NICE (National Institute for Clinical Excellence);2013.

2. De Meur G, Gottcheiner A. The Logic and Assumptions of MDSO–MSDO Designs In: Byrne D, Ragin C, eds. *The SAGE Handbook of Case-Based Methods* London: Sage; 2009.

3. Dale KS, Mann JI, McAuley KA, Williams SM, Farmer VL. Sustainability of lifestyle changes following an intensive lifestyle intervention in insulin resistant adults: Follow-up at 2-years. *Asia Pacific journal of clinical nutrition.* 2009;18(1):114.

4. Hartmann-Boyce J, Johns D, Aveyard P, et al. Managing overweight and obese adults: update review. The clinical effectiveness of long-term weight management schemes for adults (Review 1a). 2013;London UK: National Institute for Health and Care

5. NHS Centre for Reviews and Dissemination. *Systematic Reviews: CRD’s Guidance for Undertaking Reviews in Healthcare.* York, UK: Centre for Reviews and Dissemination;2008.
